# Melatonin as a Potential Adjuvant Treatment for COVID-19 beyond Sleep Disorders

**DOI:** 10.3390/ijms22168623

**Published:** 2021-08-11

**Authors:** Adam Wichniak, Aleksander Kania, Mariusz Siemiński, Wiesław Jerzy Cubała

**Affiliations:** 1 Third Department of Psychiatry and Sleep Medicine Center, Institute of Psychiatry and Neurology, 02-957 Warszawa, Poland; 2 Department of Pulmonology, 2nd Department of Internal Medicine, Faculty of Medicine, Jagiellonian University Medical College, 31-008 Kraków, Poland; aleksanderkania@interia.pl; 3 Department of Emergency Medicine, Faculty of Medicine, Medical University of Gdańsk, 80-214 Gdańsk, Poland; msiem@wp.pl; 4 Department of Psychiatry, Faculty of Medicine, Medical University of Gdańsk, 80-214 Gdańsk, Poland; cubala@gumed.edu.pl

**Keywords:** COVID-19, melatonin, physiological effects, disease progression, treatment

## Abstract

Melatonin is registered to treat circadian rhythm sleep–wake disorders and insomnia in patients aged 55 years and over. The essential role of the circadian sleep rhythm in the deterioration of sleep quality during COVID-19 confinement and the lack of an adverse effect of melatonin on respiratory drive indicate that melatonin has the potential to be a recommended treatment for sleep disturbances related to COVID-19. This review article describes the effects of melatonin additional to its sleep-related effects, which make this drug an attractive therapeutic option for treating patients with COVID-19. The preclinical data suggest that melatonin may inhibit COVID-19 progression. It may lower the risk of the entrance of the SARS-CoV-2 virus into cells, reduce uncontrolled hyper-inflammation and the activation of immune cells, limit the damage of tissues and multiorgan failure due to the action of free radicals, and reduce ventilator-induced lung injury and the risk of disability resulting from fibrotic changes within the lungs. Melatonin may also increase the efficacy of COVID-19 vaccination. The high safety profile of melatonin and its potential anti-SARS-CoV-2 effects make this molecule a preferable drug for treating sleep disturbances in COVID-19 patients. However, randomized clinical trials are needed to verify the clinical usefulness of melatonin in the treatment of COVID-19.

## 1. Introduction

The pandemic caused by the coronavirus disease 2019 (COVID-19) increased the awareness of several health-related factors that reduce the risk of infection and decrease the likelihood of a severe course of the disease. A good sleep quality is identified among those factors, as sleep plays numerous essential functions, promoting health, regenerative, and immunomodulatory processes. Thus, a poor sleep quality impairs the immune system and increases the organism’s susceptibility to infection [1]. The empirical evidence shows a link between a short sleep duration (below 6 h) before viral exposure and increased (over four times) susceptibility to developing a common cold after the administration of nasal drops containing the rhinovirus [2]. A short sleep duration was demonstrated to negatively affect antibody responses to influenza and hepatitis B vaccinations [3,4]. Taking into account that a good sleep quality also plays a crucial role in the regulation of emotions [5], the clinical data strongly support the role of educational activities and interventions aimed at improving sleep quality as essential strategies to protect society against COVID-19 and help people to cope with the stress related to the COVID-19 pandemic.

The need for sleep-improving interventions to achieve better health outcomes is further supported by epidemiological data showing that sleep disorders belong to the most common health problems in developed countries [6]. In the European guidelines for treating insomnia, it has been estimated that the average prevalence of this disorder in Europe is as high as 10% [7]. The number of people who have transient insomnia is even higher, and the percentage of people presenting limited insomnia symptoms or sleep complaints may be as high as 50% [8]. The COVID-19 pandemic is associated with a deterioration of sleep quality in society. Early data from China showed that 76.7% of the medically isolated population reported a difficulty falling asleep at least once over one week [9]. The prevalence of insomnia symptoms among self-reported isolation and non-reported isolation groups was significantly lower (51.0% and 42.3%, respectively) [9]. This observation was confirmed in later studies from other countries. The data from a representative sample of the general population in France showed that 74% of the participants had trouble sleeping, compared to 49% in the last general population survey [10]. As insomnia disorder is generally more common in women than in men, more severe sleep problems were more prevalent in females (31%) than in males (16%). Unexpectedly, young people (aged 18–34 years) reported sleep problems more frequently (79% vs. 72%) than older adults (aged 35 or older), and an alarmingly high population (16%) reported sleeping pill use during the last 12 months, since the start of the COVID-19-related lockdown [10]. Data on the negative impact of COVID-19 confinement on sleep quality have been expanded by a recent publication on neurological and psychiatric outcomes in 236,379 COVID-19 survivors during the six months following a COVID-19 diagnosis. New cases of insomnia were observed in 2.53% of patients diagnosed with COVID-19. The incidence of insomnia was lowest (2.23%) in non-hospitalized patients and highest (4.24%) in patients who required admission to the intensive therapy unit (ITU) and in those who developed delirium and other altered mental states (5.05%) [11].

Experts in cognitive-behavioral therapy proposed several operationalized behavioral interventions to counteract the deterioration of sleep quality related to the COVID-19 pandemic. Psychological treatment aims to help people cope with the stressors associated with the COVID-19 pandemic, promote sleep regulation, focusing on homeostatic sleep drive and circadian sleep rhythm, and change negative cognitions related to the bed and the bedroom from not sleeping into reformulated cognitions linked with good quality sleep. These interventions address, first of all, the negative sleep-related consequences of home confinement due to the COVID-19 pandemic, reducing physical activity and social interactions and disturbing sleep rhythm [12]. However, despite their efficacy and general recommendations that hypnotics should be avoided, because their effectiveness is questionable and can have some side effects if taken long-term [7,12], many patients still require pharmacological treatment. One of the drugs frequently used as an alternative to hypnotics to treat sleep disturbances is melatonin, which is widely available in many countries as an over-the-counter medicine.

Melatonin is a derivative of tryptophan, synthesized mainly in the pineal gland. Its synthesis is characterized by a regular circadian rhythm, with a peak concentration in plasma reached in the night hours. The intensity of melatonin production is related to exposure to light, with an increase of its synthesis in the dark part of the day [13]. Thus, melatonin serves mainly as a photoperiodic switch, influencing the activity of the suprachiasmatic nucleus and facilitating falling asleep in humans [14]. The main indication regarding the use of melatonin is for the treatment of circadian sleep–wake rhythm disorders. In this indication, melatonin has a clear advantage over hypnotics [15,16]. However, it is not generally recommended for treating insomnia [7] and should be considered only in insomnia patients aged 55 or over, mainly due to its high safety, compared to hypnotics, and the decline in melatonin production seen with age [17].

The high safety of melatonin treatment, the essential role of social rhythm changes, negatively influencing circadian sleep rhythm and causing deterioration of sleep quality during COVID-19 confinement, the lack of dependence risk of melatonin treatment, and the fact that melatonin has no adverse effect on respiratory drive have caused researchers and clinicians to consider melatonin not only as a recommended treatment for sleep disturbances, but also as an adjuvant treatment for COVID-19.

This review article aims to describe additional effects of melatonin, on top of its sleep-related effects, that make this drug an attractive therapeutic option for treating sleep disturbances during the COVID-19 pandemic and patients with COVID-19.

## 2. COVID-19 and Potential Clinical Applications of Melatonin

COVID-19 is a disease resulting from an infection with the severe acute respiratory syndrome coronavirus-2 (SARS-CoV-2). The most frequent symptoms include fever, fatigue, anosmia with ageusia, coughing, and dyspnea, which usually occur around 3–5 days from the initial infection [18]. The spectrum of the disease is broad, ranging from true asymptomatic or paucisymptomatic infection to fatal disease complications [19]. The disease affects not only the respiratory system, but also the nervous, digestive, and circulatory systems. It may also affect the kidneys, bone marrow, and other organs [20,21]. However, respiratory tract involvement is the most common cause of death among COVID-19 patients. The mechanism of death is almost always associated with acute respiratory failure and, finally, acute respiratory distress syndrome (ARDS) [22].

To date, no effective drug against SARS-CoV-2 has been approved for human use. However, evidence-based medicine permits us develop treatment guidelines [23]. The optimal strategies based on early recognition and intervention seem to be the best option [24].

The search for new drugs and the study of the properties of the already known molecules in the fight against the COVID-19 pandemic is the subject of intensive research. While no empirical data supported by clinical trial results are available on the usefulness of melatonin in the treatment of COVID-19, the general knowledge on melatonin pharmacodynamics may facilitate its hypothesized use in the treatment of COVID-19 and promote clinical research [25]. The effects of melatonin in COVID-19 may include both the preclinical period, the acute phase of the disease, acute phase complications, and late complications of COVID-19 [26,27].

Particular attention should be paid to the possibility of using melatonin in connection with vaccination [28]. The use of melatonin in the period before falling ill could also potentially reduce the severity of the disease and increase the percentage of people who are asymptomatic and suffer a mildly advanced disease. Furthermore, the effects of melatonin may be of particular importance in people with COVID-19 lung involvement. The protective effects of melatonin on lung injury have already been reviewed [29]. Notably, the concepts of melatonin’s action are based not only on its sleep-regulating effect, but also on the described anti-inflammatory, antioxidant, and immunomodulatory properties (Table 1).

## 3. Physiological Effects of Melatonin Inhibiting COVID-19 Progression

Apart from its crucial function in sustaining sleep–wake rhythm, melatonin is suggested to play a role in other physiological and pathological processes, although whether this is a direct effect of this neurohormone or indirect consequence of a melatonin-driven fluctuation in the diurnal activity of the organism is still debated. Nevertheless, a significant role of melatonin is postulated, for instance, in neurodegenerative processes [32], metabolism control [33], cardiovascular disorders [34], and (which has a special meaning in the context of this review) in inflammatory diseases [35]. The last connection is additionally underlined by the fact that melatonin is produced by cells of the immune system (mononuclear cells, lymphocytes, and macrophages), stimulated by bacterial antigens [36]. Therefore, it is widely postulated that melatonin possesses anti-inflammatory potential, which may be beneficial in bacterial or viral infections.

There is a line of evidence proving that melatonin may exert anti-inflammatory action, which could hypothetically be useful in the treatment of inflammation evoked by SARS-CoV-2 infection. In this context, the following features of melatonin have specific significance: interaction with the renin-angiotensin system, suppression of proinflammatory cytokines, and antioxidant properties.

### 3.1. Renin-Angiotensin System

The renin-angiotensin system (RAS) is well known to regulate the cardiovascular system, but it is also involved in other physiologic phenomena, e.g., the development of inflammatory response. Recently, RAS gained special attention due to its role in SARS-CoV-2 infection. The virus enters the host’s cells by binding the virus’s spike protein with angiotensin-converting enzyme 2 (ACE2), localized on the cell’s membrane [37]. This explains why, hypothetically, SARS-CoV-2 causes an injury to organs rich in ACE2 (e.g., the lungs, cardiovascular system, and liver) and why in patients with a cardiovascular comorbidity related to a higher activity of RAS, there is an increased risk of a severe course of COVID-19.

The abovementioned finding leads to the hypothesis that reducing the RAS activity, with a lower expression of ACE2 on the cell’s membrane, may lower the virus’s potential to invade the cell. The antagonism between melatonin and RAS was described previously, and the available clinical observations allowed researchers to coin the term, “angiotensin–melatonin axis” [38]. On the sub-cellular level, it was discovered that the surface expression of ACE2 is regulated by calmodulin [39]. Melatonin can bind calmodulin and inhibit its action [40,41]. Therefore, it is plausible that melatonin, through the inhibition of calmodulin, may reduce the expression of ACE2 and limit viral invasion, although this was not proven clinically.

### 3.2. Cytokine Suppression

The severity of COVID-19 is not a consequence of the toxicity of the virus itself but of the exaggerated response of the host’s immune system, a hyper-inflammation driven by the overproduction of proinflammatory cytokines, which is called a “cytokine-storm” [42]. The recognition of the viral molecular patterns (pathogen-associated molecular patterns, PAMPS) by pattern-recognizing receptors (PRRs) [43] activates PRRs and leads to the production of transcription factors, which, in turn, promotes the production of proinflammatory cytokines. One of the most important transcription factors is the nuclear factor kappa B (NF-kB). The presence of NF-kB and other transcription factors leads to an increased expression of proinflammatory cytokines. Among them, the most significant are interleukin 1 (IL-1), interleukin 6 (IL-6), and tumor necrosis factor alpha (TNF alpha) [44]. The cytokines cause a migration of immune cells to the infection site, damage to the vascular barrier, cell apoptosis, and, further, a fulminant development of inflammation, leading to multiorgan failure and death. Therefore, reducing the concentration of circulating proinflammatory cytokines is a way to inhibit the COVID-19-related cytokine storm.

Melatonin is proven to reduce the activity of cytokines. Qin et al. [45] found that melatonin can reduce the expression and activity of matrix metalloproteinase 9 (MMP9) in human cells stimulated by interleukin 1 beta by suppressing NF-kB activation. It was also shown, in another study, that melatonin suppresses the production of a proinflammatory cytokine (interleukin 8) in human pulmonary fibroblasts stimulated with acrolein [46]. There are also some limited data from human studies. A randomized clinical trial showed that treatment with melatonin reduced proinflammatory cytokines (IL-1 beta, IL-6, and TNF alpha) in patients with chronic kidney disease [47].

Furthermore, Alamili et al. [48] observed that melatonin administration resulted in a reduction of some proinflammatory cytokines (IL-1 beta) in a human model of sepsis. It was also shown that this neurohormone suppresses the activation of NF-kB [49,50]. Secondly, through the up-regulation of nuclear factor erythroid 2-related factor 2 (Nrf-2), melatonin causes a reduction in the activity of proinflammatory cytokines, such as Il-1, Il-6, and TNF [50,51]. These data, from in vitro studies and from animal models, give hope that melatonin may help stop COVID-19-related hyper-inflammation, though data from human studies are still needed.

### 3.3. Free Oxygen Species Scavenging

Melatonin is supposed to act as an antioxidant agent. Due to its chemical structure, it may serve as a scavenger of radical oxygen and nitrogen species [52]. Apart from its direct antioxidant action, melatonin can increase the expression of the genes of antioxidant enzymes, such as superoxide dismutase or glutathione peroxidase, and then increase their activity [53]. It was proven that melatonin simultaneously exerts a protective effect against oxidative damage to DNA through the activation of DNA repairing processes [54]. It is also crucial that the metabolites of melatonin exert antioxidant potential. 6-hydroxymelatonin and 4-hydroxymelatonin are capable of repairing damage to DNA caused by oxidative stress [55]. The production of free radicals is a natural consequence of hyper-inflammation and the activity of proinflammatory cytokines. Their presence is responsible for massive damage to tissues resulting from inflammation caused by SARS-CoV-2 infection. Therefore, keeping in mind that all data come from in vitro experiments or animal model studies, the hypothesis that melatonin acts as a protective antioxidant factor in COVID-19 becomes plausible.

### 3.4. Melatonin in Severe Inflammation

All the abovementioned anti-inflammatory features of melatonin made plausible the hypothesis that it has a therapeutic role in generalized inflammation and sepsis. Indeed, in a series of experiments performed on animal models of sepsis, melatonin was proven to reduce the inflammatory response and protect tissues from oxidative injury [56,57,58]. The literature review by Biancatelli et al. [59] concluded with the suggestion that melatonin can be used in patients with severe sepsis and septic shock, albeit while emphasizing the scarce clinical evidence. So far, it was proven in an ex vivo study of blood taken from human volunteers and treated with bacterial lipopolysaccharides that melatonin causes a reduction of oxidative stress and the proinflammatory activity of cytokines [60]. Certainly, these data are not sufficient to include melatonin in the antiseptic or anti-inflammatory armamentarium, though it opens a promising area of clinical studies (Table 2).

It must be remembered that severe inflammation/sepsis is directly related to severe mitochondrial dysfunction. It has been shown that melatonin protects mitochondria and restores their proper function through the normalization of adenosine triphosphate (ATP) synthesis, reversal of the inhibition of mitochondrial enzyme complexes, and restoration of the mitochondrial membrane potential [61,63,64].

## 4. Melatonin—Hypothesized Use in COVID-19—Impacting the Burden of the Disease

**Figure 1:**
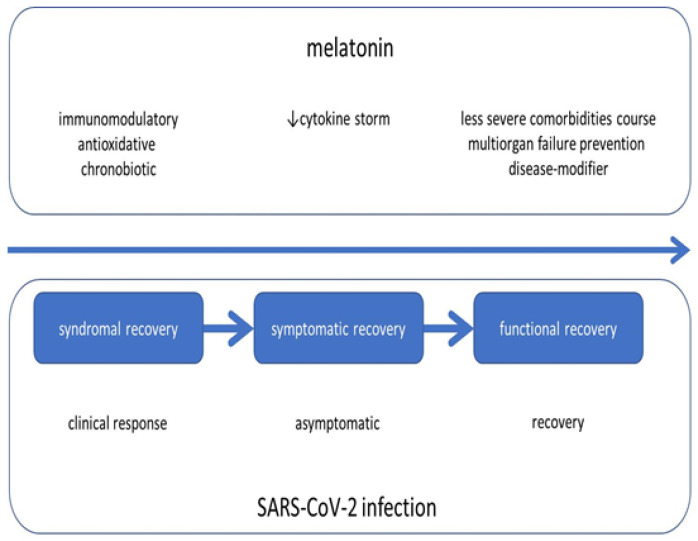
SARS-CoV-2 infection and adjuvant actions of melatonin.

Melatonin exhibits a range of actions in humans that may demonstrate therapeutic potential in COVID-19 patients associated with counteracting the disease onset, course, and resolution (Figure 1). Melatonin acts as a chronobiotic agent, with antioxidant, anti-inflammatory, and immunomodulatory properties [65]. Its use in COVID-19 may involve: SARS-CoV-2 infection prevention, antiviral activity, prevention of circadian disruption, antioxidant/anti-inflammatory/immunomodulatory activity, disease-modifying properties in subjects with comorbidities, and acting as an adjuvant to potentiate anti-SARS-CoV-2 vaccines [31,65].

### 4.1. Melatonin—Adjuvant in COVID-19 Clinical Management

Melatonin is reported to promote the activity of drugs used in COVID-19 prevention and treatment. It is discussed as a protective drug used in combination treatments with prednisone/methylprednisolone for relieving edema and its consequences, particularly SARS-CoV-2-related pneumonia, lung dysfunction, and lung failure. Moreover, melatonin used as an immunomodulatory agent in combination with nucleotide analogs, e.g., ribavirin or remdesivir, may promote the antiviral activity of the drugs [25].

In line with the preclinical evidence of melatonin ameliorating the course of septic shock with the related renal injury, septic cardiomyopathy, and liver injury, it exerts a neuroprotective effect [66]. In humans, deep sedation and delirium are associated with an increased long-term mortality [31,66]. Melatonin may be considered a safe and effective strategy for preventing sleep disturbances and delirium in COVID-19 inpatients requiring high-intensity treatment procedures, including intensive care management. Melatonin appears to reduce the incidence of delirium and its severity by reducing its risk factors related to disturbed sleep and, perhaps, partly preventing the accumulation of immune system dysfunction [31]. Moreover, melatonin use reduces sedative prescriptions in inpatients and ameliorates patients’ general well-being, reducing the reported pain, agitation, and anxiety, which are related to an overall treatment improvement, less frequent complications, and patients’ well-being [66].

Above all, the safety and tolerability profile is favorable for melatonin use in humans with a low incidence of mild adverse effects mostly associated with sleep structure alternations [31,67].

### 4.2. Melatonin—Anti-SARS-CoV-2 Vaccine Adjuvant

Melatonin is known to enhance the immune response to vaccines. Its use may act as an adjuvant in anti-SARS-CoV-2 vaccination, increasing the strength and duration of the immunity acquired [28,67]. Melatonin counteracts immunosuppression, including the one resulting from corticosteroid use, increasing the IgG-expressing B cells, IgG antibody response, and peripheral blood CD4+ T lymphocytes. The latter is relevant to the observation of a vigorous response of CD4+ T cells to the spike protein in subjects recovering from COVID-19 [26,27,67].

Melatonin may be particularly useful in the elderly population, males in particular, enhancing immune responses and reversing age-related immune decline. While the evidence is unclear, it seems that sleep quality deteriorates with age, producing an immune system impairment. Thus, in line with a focus on sleep quality prior to vaccination, the melatonin chronobiotic effect may be beneficial and shall be considered two weeks before vaccination, ensuring vaccination is performed at a moment of optimal sleep conditions. In addition to the immunomodulatory and antioxidant properties of melatonin, its chronobiotic properties may provoke physicians to administer it at four weeks after vaccination as a vaccination enhancer [67].

### 4.3. Transformative Treatment with Melatonin in COVID-19

Melatonin use in COVID-19 may represent a concept of transformative intervention that modifies the risk factors, onset, course, resolution, and prophylaxis of the disease at its different stages from a short-term and long-term perspective. Melatonin may impact and modify the state and trait characteristics of the illness and address the predisposing factors, recent characteristics, and current state. The use of melatonin as an adjuvant in COVID-19 clinical management and vaccination planning and management is particularly interesting [25,31].

## 5. Protective Role of Melatonin in Lung Diseases

The fatal consequence of SARS-CoV-2 infection is pneumonia, resulting in respiratory insufficiency, and probably the most prominent long-term effect of COVID-19 is disability resulting from an injury of the lungs. Therefore, it is noteworthy that melatonin in animal models appears to be a therapeutic/protective agent in acute and chronic pulmonary diseases.

From a clinical point of view, the crucial area for melatonin use in the treatment of severe COVID-19 is related to its action in preventing diffuse alveolar damage (DAD). DAD is considered the histological hallmark for the acute phase of acute respiratory distress syndrome (ARDS) [68]. Numerous experimental studies in animal models have shown a beneficial effect of melatonin on lung tissue [29]. It was shown that melatonin improved the histopathology of pulmonary contusion and distant organs. Ozdinc et al. [69] found that in rats treated with melatonin, histopathological changes resulting from pulmonary contusion were less severe than in non-treated animals. The authors suggested that this is the effect of the antioxidant properties of the neurohormone [69]. Moreover, melatonin reduces leukocyte and macrophage infiltration, interstitial hemorrhages, epithelial desquamation in bronchioles and alveoli, and an intra alveolar edema [70].

The other aspect is the ability of melatonin to decrease lung ischemia/reperfusion injury (LIRI). Chiu et al. [71] revealed that melatonin significantly diminished pulmonary microvascular permeability and attenuated the lipid peroxidation in the lungs. In fish, melatonin significantly inhibited the NF-kB expression and downregulated the activity of nuclear factor-erythroid-2–related factor 2 synthesis in hepatic ischemia/ reperfusion-induced lung injury [72].

In COVID-19 patients, a reduction in pulmonary gas exchange was observed due to an impaired surfactant function resulting from lipid peroxidation due to neutrophil infiltration. Furthermore, in vitro experiments with melatonin have shown that melatonin can bind to surfactant lipids and reduce their peroxidation [73,74]. This creates additional areas for its beneficial effects.

Pulmonary embolism is another frequent pathomechanism of respiratory failure in COVID-19 [75]. It is often the direct cause of death. Considering the evidence that melatonin affects the coagulation processes [76], it can be expected to have a beneficial effect in this area.

Treatment of COVID-19 respiratory failure often requires the use of positive airway pressure before intubation [77]. This kind of treatment increases the risk of ventilator-induced lung injury (VILI) and pneumothorax [78]. It was observed that melatonin decreases VILI by increasing the anti-inflammatory response, despite causing an unexpected increase in oxidative stress [79].

Saving a patient’s life in an acute period of infection does not put an end to the challenges associated with COVID-19. Even after a mild illness, many patients experience symptoms associated with infection with the virus for many months [80]. COVID-19 and particularly lung damage can finally lead to chronic life-limiting complications [81]. Post-COVID-19 pulmonary fibrosis, which may lead to chronic respiratory failure, is the best example of this [82]. Until now, no drug protecting against post-COVID-19 pulmonary fibrosis has been reported. The use of antifibrotic drugs in idiopathic pulmonary fibrosis is still being researched in connection with COVID-19. The application of melatonin in this context can be an additional area for its use. Zhao et al. [83] showed that melatonin protected against lung fibrosis by regulating the Hippo/YAP pathway. The Hippo signaling pathway is an intrinsic, central mechanism that restricts organ size [84]. This pathway regulates the transcription of target genes involved in cell growth, proliferation, and survival. The yes-associated protein (YAP) is a crucial transcription factor in the Hippo signaling pathway and is negatively regulated by this pathway. Dephosphorylated YAP enters nuclei and interacts with transcriptional enhancer factor domain family proteins to induce the expression of the target gene [85]. Melatonin attenuates lung fibrosis by inhibiting the expression and activation of YAP1 by binding to MT1 and MT2 Melatonin Receptors [83].

Moreover, melatonin was proven to be helpful in post-radiation lung fibrosis [86]. It was also shown to inhibit apoptosis and reduce inflammation in animal models of chronic obstructive pulmonary disease (COPD) [87,88]. Melatonin also attenuates fibrotic processes in COPD [89]. Hosseinzadeh recently published a review of the evidence for a possible protective role of melatonin in idiopathic pulmonary fibrosis [90]. It was also shown that melatonin exerts a beneficial effect in experimental models of asthma by remodeling airways [91] and suppressing inflammation [92]. All of the abovementioned discoveries were recently described in animal models. Therefore, they cannot be directly translated to human medicine. Nevertheless, they are sufficient to warrant the proposal of melatonin as a candidate “pneumo-protective” drug in viral infections.

## 6. Discussion

As a considerable simplification, the pathologic cascade of COVID-19 consists of the following events: the entrance of the SARS-CoV-2 virus into the cell through binding with membrane angiotensin-converting enzyme 2, further incorporation of the virus, recognition of the virus through pattern-recognizing receptors, activation of transcription factors (such as NF-kB), further production of proinflammatory cytokines, an inflammatory response, conversion to uncontrolled hyper-inflammation (the “cytokine storm”), progression of multilocal damage to tissues, and in most severe cases, respiratory insufficiency and multiorgan failure, leading to death or some form of chronic disability (e.g., pulmonary damage, resulting from fibrotic changes within the lungs).

From the data presented in the previous paragraphs, a conclusion may be drawn that melatonin has the potential to attenuate this fatal cascade at all levels. First, the pineal neurohormone reduces the activity of the renin-angiotensin system, potentially reducing the expression of ACE2. Second, it inhibits the function of transcription factors and the consequent production and activity of proinflammatory cytokines, potentially preventing the development of hyperinflammation. Finally, by acting against oxidative damage, the apoptosis of cells, and the fibrotic changes of tissues, it may protect organs from COVID-19-related chronic dysfunction (Figure 1).

While all the abovementioned facts are speculatory, based on evidence from in vitro or animal experiments, melatonin has received significant attention from the beginning of the COVID-19 pandemic. However, the main disadvantage of this theory is that it has not yet been supplemented by convincing clinical trials.

### Clinical Data of the Effects of Melatonin in COVID-19

Clinical data about the efficacy of melatonin in patients struggling with COVID-19 are scarce. An in silico study on the theoretical model, seeking the most suitable anti-SARS-CoV-2 drug, found that melatonin could be a promising candidate [93]. The first observational clinical data from Ramlall et al. [94] (publication did not go through peer review) reported the relation between the intake of various drugs and survival rates among intubated COVID-19 patients. The data came from over 790 patients. The authors found that subjects taking melatonin had a higher probability of a positive outcome. Regardless of the overall optimistic resonance of this study, it has some limitations. Firstly, it was a retrospective study. Therefore, it did not assess any specific treatment protocol with melatonin. Secondly, the authors did not present data on the reason for the melatonin intake nor its dosage. Finally, the relationship between melatonin and a positive outcome is not so obvious, as a similar effect was observed in patients taking other drugs with sleep-inducing properties: benzodiazepines, quetiapine, and trazodone. This suggests that sedation- or drug-induced increased sleep may be responsible for the positive effect [94].

There is one low-quality study directly examining the therapeutic effect of melatonin. Castillo et al. [95] described the effect of a high dose (36–72 mg per day) of melatonin, given orally to patients with a clinical suspicion of COVID-19. The authors performed this therapy in 10 cases, among which seven were confirmed by PCR test to be COVID-19 positive. The treatment effect was positive: none of the patients died; moreover, none of them needed mechanical ventilation. At the same time, in the center performing the study, the COVID-19 mortality was 35.5%, and 20.6% required mechanical ventilation. Naturally, this study has significant disadvantages: (a) the treated group was tiny; (b) there were no clear inclusion/exclusion criteria; (c) the study was not blinded; and (d) a control group was not implemented. These facts prevent us from formulating any opinions on melatonin effectiveness and safety based on this study [95].

The protocols of some clinical studies on melatonin usage have been published. They aim to verify the efficacy and safety of various doses of melatonin given in diverse stages of the disease [53,96]. According to www.clinicaltrials.gov (accessed on 15 May 2021), there have been ten registered clinical trials on melatonin in COVID-19, with one of them withdrawn and one completed.

As shown in the above paragraphs, the biological properties of melatonin, its physiologic interactions with the inflammatory system, and its relation to the course of SARS-CoV-2 infection allow for hope that this sleep-inducing neurohormone may become a helpful treatment in COVID-19. In particular, in considering the potential use of melatonin in the treatment of COVID-19, its synergy of action with other drugs used in the treatment of these patients should be taken into account [54]. Perhaps combining melatonin with other specific molecules would prove particularly beneficial. It is also worth looking for a specific phenotype of a patient with COVID-19 that would benefit from its use. It would be optimal to have an objective marker that predicts a good response to melatonin use. Little is known about the protective effects of melatonin on something other than respiratory systems and organs damaged in COVID-19. We also do not know the optimal dose of melatonin administration today to support COVID-19 therapy and the phase of the disease in which the administration of melatonin would be most effective. Finally, the evidence of the positive effects of melatonin relating to disturbed circadian sleep rhythm [15,16] and insomnia in subjects aged 55 or over [17] cannot be simply transferred and used in the treatment of COVID-19-related sleep disorders. In particular, patients with sleep disturbances related to the COVID-19 pandemic associated with home confinement have to be distinguished from patients with insomnia associated with SARS-CoV2-infection. The latter group may require a different dose of melatonin from that described for the treatment of circadian sleep–wake rhythm disorders and insomnia.

## 7. Conclusions

The high safety profile of melatonin, either alone or in a combined therapy, and its potential anti-SARS-CoV-2 effects make this molecule a first-line drug that should be considered for the treatment of sleep disturbances in COVID-19 patients. However, to verify the clinical usefulness of melatonin in the treatment of COVID-19, it is necessary to wait until the end of the clinical trials.

## Figures and Tables

**Table 1:** Potential effects of melatonin on the course of COVID-19 (modified from: [27,29,30,31]).

COVID-19 Phase	Effect of Melatonin Treatment	Recommended Dose
Prophylaxis	Circadian sleep–wake rhythm disorder treatment	0.5–5 mg
	Insomnia treatment Adjuvant to anti-SARS-CoV-2 vaccines	2–5 mg ^1^ 0.5–2 mg ^2^
Early infection phase/mild clinical symptoms	Improvement of sleep quality Inhibition of viral invasion Free oxygen species scavenging Cytokine suppression	2–12 mg ^3^
Pulmonary phase/severe clinical symptoms	Improvement of sleep quality Anti-inflammatory effects Reduction of “cytokine-storm” Protection of lungs and tissues from oxidative injury Risk reduction of ventilator-induced lung injury	2–12 mg ^3,4^
Post-infection period	Improvement of sleep quality Stabilization of the disrupted circadian sleep rhythm Antioxidant properties Reduction of post-covid pulmonary fibrosis	0.5–5 mg

^1^ Preferred prolonged-release form [17]. ^2^ high doses should be avoided, as they may decrease the immune response [28]. ^3^ The optimal dose is unknown due to a lack of randomized clinical trials. ^4^ Doses as high as 400 mg per day have also been suggested [30].

**Table 2:** Current data on the physiological effects of melatonin, which may be beneficial in bacterial or viral infections.

In Vitro Studies	Animal Models	Human Studies
Inhibition of calmodulin [40]	Regulation of anti/proinflammatory cytokines balance [56]	Inhibition of IL-6, IL-1, TNF-alpha [47]
Inhibition of metalloproteinases [45]	Restoration of ATP production [61]	Reduction of the concentration of IL-1 beta [48]
Reduction of the production of IL-8 [46]	Organ protection [58]	Reduction of lipid peroxidation [62]
